# Evidence of Pollinators Foraging on Centipedegrass Inflorescences

**DOI:** 10.3390/insects11110795

**Published:** 2020-11-13

**Authors:** Shimat V. Joseph, Karen Harris-Shultz, David Jespersen

**Affiliations:** 1 Department of Entomology, University of Georgia, Griffin, GA 30223, USA; 2 Crop Genetics and Breeding Research Unit, USDA-ARS, Tifton, GA 31793, USA; karen.harris@usda.gov; 3 Department of Crop and Soil Science, University of Georgia, Griffin, GA 30223, USA; djesper@uga.edu

**Keywords:** *Eremochloa ophiuroides*, *Lasioglossum*, sweat bee, *Bombus*, bumble bee, turfgrass, spikelet

## Abstract

**Simple Summary:**

Turfgrasses are generally considered devoid of pollinators, as turfgrasses are often described as being only wind-pollinated. Centipede grass is a popular turfgrass grown in the southeastern USA. Centipede grass produces a large number of inflorescences from August to October each year. In a recent study, honeybees were found to collect pollen from centipede grass. However, it is not clear whether other pollinators are attracted to centipede grass inflorescences and actively forage them. Thus, the aim of the current study was to document the pollinators that foraged on centipede grass inflorescences. Pollinators visiting centipede grass were sampled using (1) a sweep net when actively foraging on an inflorescence; (2) blue, white and yellow pan traps; and (3) malaise or flight-intercept traps. Sweat-, bumble- and honeybees were captured while actively foraging on the centipede grass inflorescences. In the pan and flight-intercept traps, more sweat-bees were collected than honey- or bumblebees. We also captured hoverflies in the samples. The adult hoverflies consumed pollen during flower visits. This research is a first step toward developing bee-friendly lawns. The data also imply that proper caution should be exercised to preserve bee habitat and encourage bee foraging.

**Abstract:**

Turfgrasses are commonly used for lawns and as recreational surfaces in the USA. Because grasses are largely wind-pollinated, it was thought that pollinators would not forage on turfgrasses. Centipede grass (*Eremochloa ophiuroides* (Munro) Hack) is a warm-season turfgrass widely used in the southeastern USA. Centipede grass produces spike-like inflorescences from August to October, and little is known about whether pollinators utilize those inflorescences as pollen resources. Thus, the objective of the current study was to identify the pollinators foraging on centipede grass inflorescences. Pollinator samples were collected by (1) sweeping the insects actively foraging on centipede grass inflorescence for 30 min, (2) deploying pan traps for 24 h and (3) deploying malaise traps for 7 d. In the sweep samples, *Lasioglossum* spp., *Bombus* spp., *Apis* spp., *Melissodes* spp. and *Augochlorella* spp. were collected from centipede grass inflorescences. Syrphid flies were also collected in the sweep samples. The pan and malaise traps collected mostly *Lasioglossum* spp. The results imply that there is a critical need to conserve bee habitats and adopt nondisruptive lawn practices. Additionally, this new knowledge lays the foundation for future research to enhance our understanding of bee and syrphid behavior and the selection of host traits for improving bee foraging.

## 1. Introduction

Pollinators utilize floral resources available to them in green spaces [1,2,3,4,5]. The incidence and abundance of pollinators are driven by available floral resource communities in the landscape [6,7,8,9]. However, lawns in the USA are typically maintained aiming for a green monoculture free of weeds or other flowering plants. Turfgrasses are regularly trimmed and mowed at regular intervals. In public and residential lawns, patches of flowering weeds, such as clovers or dandelions, are common, and these weeds often provide supplemental nectar and pollen for foraging bees [4]. A diverse group of pollinators have been documented to occur in urban and suburban lawns in southern Connecticut [10], New York [11], Massachusetts [4], and Georgia [12]. In addition, ground-nesting bees construct nests in the patches of exposed bare soil along the edges of some lawns [13].

**Figure 1:**
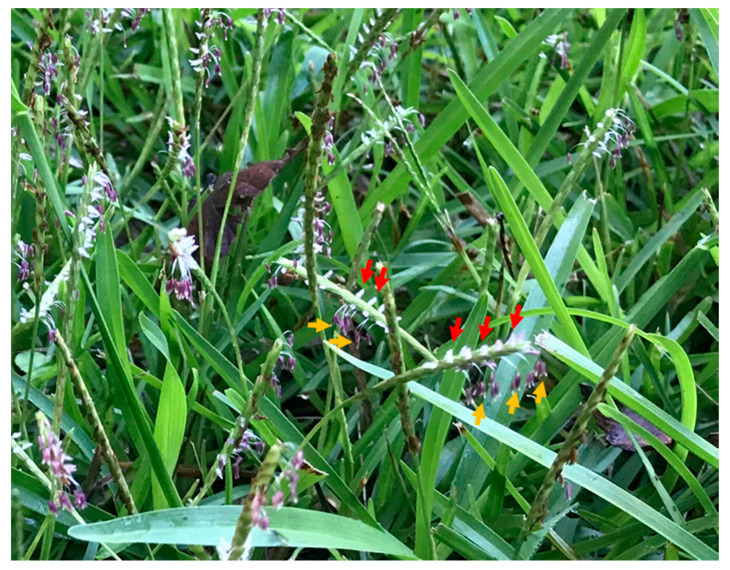
Spike-like inflorescences of centipede grass emerged 12 d after mowing in a residential lawn in Pike County, GA, USA, during September 2020. The androecium (stamens) (purple anthers and white filament) containing pollen (indicated by orange arrows) and gynoecium (carpel) (white stigma, and style), which receive pollen (indicated by red arrows) are shown in the figure. Photo credit: Shimat Joseph.

Turfgrass is an integral and inseparable element of the southeastern USA. It provides an essential green cover, enhancing the aesthetics and property value of landscapes. In Georgia, the turfgrass industry is valued at 7.8 billion USD in 2010 [14]. Among warm-season turfgrasses, centipedegrass (*Eremochloa ophiuroides* (Munro) Hack) is one of the major species found in managed landscapes in the southeastern USA, as it requires minimal management and fertilization [15]. A recent study showed that a diverse group of bee genera, mainly *Lasioglossum* spp., transit in centipede grass lawns in Georgia [12]. Centipede grass typically produces spike-like inflorescences (raceme) beginning in August in the southeastern USA. The structures of grass flowers (spikelets) include a gynoecium, androecium, lodicules, palea, and lemma [16]. The gynoecium and androecium of flowers appear from raceme (Figure 1) and support wind pollination. However, the European honeybee, *Apis mellifera* Linnaeus, has been found to forage on the inflorescences of centipede grass [17]. Of note, when the honeybees traveled from inflorescence to inflorescence, they generated biotic winds that moved the pollen significant distances, although the exact distance the pollen travelled by biotic wind is not clear. A previous survey found a diverse group of bees in unmowed lawns when centipede grass produced inflorescences [12], with *Lasioglossum* being the most abundant bee genera. To develop a bee-friendly centipede grass lawn, it is critical to document the direct foraging activity of bees on the inflorescences of centipede grass. Thus, the objectives of the current study were to determine (1) the diverse groups of pollinators that directly visit or forage on the inflorescences of centipede grass and (2) the relative abundances of pollinators foraging on centipede grass using various trapping devices.

## 2. Materials and Methods

### 2.1. Study Sites

This study was conducted on eleven centipede grass lawn sites: six sites in Spalding County, three sites in Pike County, and one site each in Meriwether County and Coweta County of Georgia, USA, from mid-August to the end of September 2020 (Table 1). The details of the lawn sites are listed in Table 1. Within the eleven centipede grass sites, eight sites were in residential lawns, and three sites were in lawns at the University of Georgia (UGA), Griffin campus, USA. Because homes are built on various plot sizes, the sizes of lawns vary. The lawns in the current study ranged from 29 to 2626 m^2^. Most of the residential lawns and two of the UGA sites were more than 1 km apart. The UGA1 and UGA3 sites were less than 1 km apart. The lawns received no pesticides, such as herbicides, fungicides, or insecticides, and approximately 0–20% of the lawn area had weeds. The number of inflorescences per 926 cm^2^ (30.48 × 30.48 cm) was quantified during each visit. The lawns were mowed at ~7.6 cm height.

### 2.2. Sampling and Evaluation

The selected lawns were mowed at 14 d intervals, and the sampling was initiated 12–13 d after mowing. The assumption was that this time interval would be sufficient for the centipede grass to put out fresh inflorescences instead of setting seed. Pollinators were sampled using three trapping methods: (1) sweep netting for 30 min during each visit; (2) three pan traps, which included yellow, blue, and white bowls left at each site for 24 h; (3) one malaise trap per site for 7 d (Table 1). For the sweep sample, a person slowly moved randomly across a lawn and when a foraging pollinator was observed on a centipede grass inflorescence (Figure 1) for 3 s, it was collected using a sweep net. A 38 cm diameter sweep net (Bioquip, Cat. #7625HS Rancho Dominguez, CA, USA) was used for sampling. The number of visits per site is listed in Table 1. The sites were visited between 09:30 and 13:00 h at approximately 12 d intervals for sweep netting. On some occasions, more than one sweep sample was collected within the 12 d intervals. Samples were not collected on rainy days or windy days. While transferring the pollinators collected in the sweep net into clear plastic bags, several of them flew away, and those lost pollinators were also recorded and were grouped as halictids (sweat bees), and apids (honey bees or bumble bees). The flies captured were all syrphid flies and were identified to the family level.

The pan traps consisted of 354.8 mL bowls (Amscan, Elmsford, NY, USA) in yellow (manufacturer’s description: yellow sunshine), blue (manufacturer’s description: bright royal blue), and white. The three pan traps were placed ~1 m apart in a triangular pattern at every site. To secure the pan traps, one bowl was first nailed through its center to the ground, and then another bowl of the same color was placed over it and secured using three binder clips. A soap solution was prepared by adding 0.5 mL of Dawn dish soap (The Procter & Gamble Company, Cincinnati, OH, USA) to 3.78 L of water, and 200 mL of the soap solution was added to each bowl. Similarly, 200 mL of the soap solution was added to each collection container of the malaise traps (BugDorm, L110 × W110 × H110 cm, SLAM Trap—Standard, https://shop.bugdorm.com/ez-malaise-trap-p-105.html). The insects collected in the sweep net were emptied into individual 3.78 mL Ziploc-style clear plastic bags. The contents of the pan and malaise trap samples were strained, emptied into plastic bags, transported to a laboratory, and temporarily stored at −4 °C in a freezer. The pollinators (bees and syrphid flies) in the plastic bags were sorted, dried after exposure to hot air using a hair dryer, and pinned for future identification. The collected bee specimens were identified to genus using keys [18]. The captured bees were grouped as halictids and apids. Because only syrphid flies were collected in the sweep samples, only these flies were sorted from the pan and malaise traps.

### 2.3. Statistical Analysis

The data on the number of bees observed (i.e., the few individuals that escaped from the sweep nets) and physically captured in the sweep samples were combined. For the analysis, each combination of site and date (visit or trap sample) was considered a replicate. The bees (halictids and apids) and syrphids captured from all three traps were combined per site/date. The data were log-transformed (ln[x + 1]) and then subjected to one-way analysis of variance (ANOVA) using a general linear model (PROC GLM) in SAS [19]. The means were separated using Tukey’s HSD test for treatment comparisons. All the statistical comparisons were considered significant at α = 0.05. An ANOVA was not performed to determine the effects of trapping methods, because the intervals of trap deployment and trapping mechanisms varied among the various traps used in the experiment.

## 3. Results

### 3.1. Pollinators Collected

**Figure 2:**
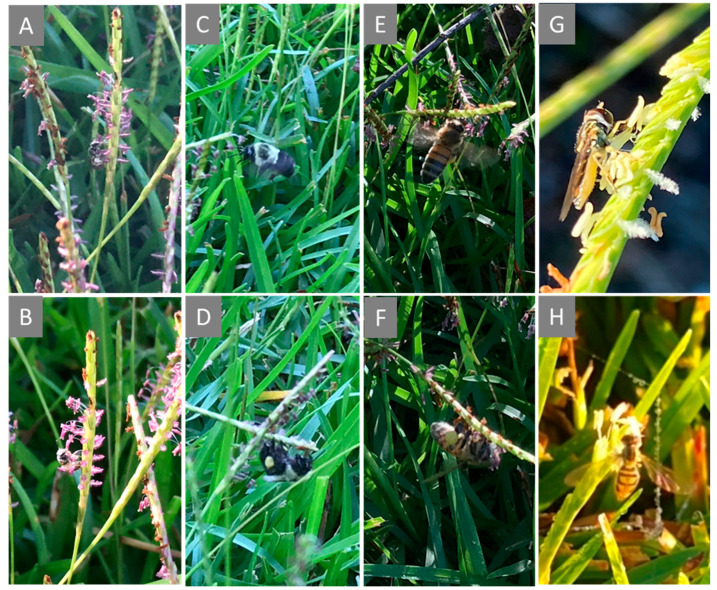
Foraging activity of ((**A**) and (**B**)) a halictid (*Lasioglossum* spp.), ((**C**) and (**D**)) a bumble bee (*Bombus* spp.), ((**E**) and (**F**)) a honey bee (*Apis* spp.), and ((**G**) and (**H**)) a syrphid fly on the spike-like inflorescences of centipedegrass in a residential lawn during September 2020. Photo credit: (**A**–**F**), Shimat Joseph; (**G**), Karen Harris-Shultz; and (**H**), Michael Purvis, USDA ARS, Tifton, GA, USA.

Ninety-three pollinators were collected from centipedegrass inflorescences in the 30 min sweep samples and most of them were *Lasioglossum* spp. (Figure 2A,B; Table 2) followed by *Bombus* spp. (Figure 2C,D) and *Apis* spp. (Figure 2E,F). *Melissodes* spp. and *Augochlorella* spp. as well as syrphid flies (Figure 2G,H) were also sampled.

In the pan traps, 41 pollinators were captured in 24 h intervals and most of them were *Lasioglossum* spp. (Table 2). Other bees captured in the pan traps were *Augochlorella* spp., *Bombus* spp. and *Melissodes* spp. Syrphid flies were also captured. In the malaise traps (flight-intercept trap), eight bees were captured over the 7 d intervals (Table 2), where most of them were *Lasioglossum* spp. and *Melissodes* spp. Syrphid flies were not collected in the malaise traps.

### 3.2. Pollinator Types Collected

**Figure 3:**
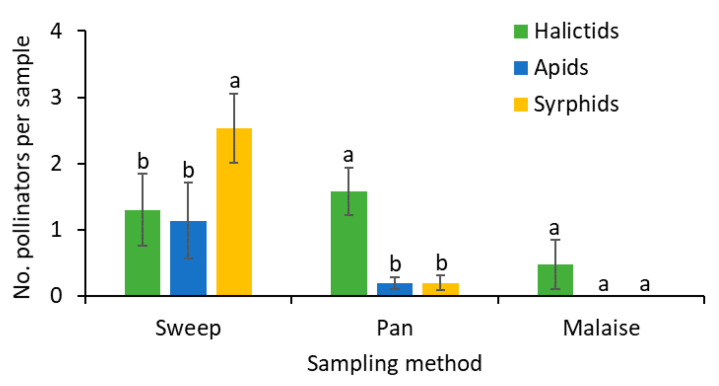
Mean (±SE) numbers of pollinators collected by three sampling methods. Within each trap type, the bars with a different letter are significantly different (Tukey’s HSD test, *p* = 0.05).

When the collected and observed pollinators were combined, the numbers of halictids and apids bees collected in the sweep samples were not significantly different, and their densities were lower than that of the syrphids (*F*_2, 55_ = 7.6; *p* = 0.001; Figure 3). In the pan traps, significantly more halictids were collected than apids or syrphids (*F*_2, 40_ = 19.1; *p* < 0.001; Figure 3). While a few halictids were collected in the malaise traps, no apids or syrphid flies were captured in these traps (*F*_2, 36_ = 2.4; *p* = 0.101; Figure 3).

## 4. Discussion

*Lasioglossum* spp., *Bombus* spp., *Apis* spp., *Melissodes* spp. and *Augochlorella* spp. were observed foraging on centipede grass inflorescences. This is the first report of *Lasioglossum* spp., *Bombus* spp., *Melissodes* spp. and *Augochlorella* spp. actively foraging on centipede grass inflorescences. Foraging by *Apis* spp. on centipede grass inflorescences has previously been reported [17]. Several bee species, including *Lasioglossum* spp., *Bombus* spp., *Apis* spp., *Melissodes* spp. and *Augochlorella* spp. were collected from centipede grass lawns [12] but were not recorded or captured while foraging on centipede grass inflorescences. In the current study, similar numbers of actively foraging *Lasioglossum* spp. (halictids) and *Bombus* spp. and *Apis* spp. (apids) were collected from centipede grass inflorescences, suggesting that a diverse group of bees forage on these inflorescences.

Adult syrphid flies consume pollen and nectar from several flowering plants [20,21]. When foraging on flowers, they directly consume pollen and nectar and transfer the pollen attached to their body and bristles among flowers [22]. In the current study, syrphid flies were observed foraging on centipede grass inflorescences, although their specific relationship with centipede grass inflorescence is not clearly understood. Previously, visitations of a syrphid fly species were observed on bamboo (*Guadua paniculata* and *G. inermis* (Family: Poaceae)) flowers [23]. Grass spikelets are not widely known to produce nectar [16], and nectar is not evident in centipede grass inflorescences. Thus, more studies are warranted to determine the foraging behavior of adult syrphids on centipede grass inflorescences. The larval stages of syrphid flies are predaceous, as they mainly consume aphids [24] and it is not clear if they transport pollen.

In a previous study [12] and the current study, *Lasioglossum* spp. were abundant in pan traps on centipede grass lawns in Georgia; this is similar to reports from lawns in the northern states [4,10,11]. In the current study, *Bombus* spp. and *Apis* spp. were collected in sweep samples, suggesting that they occur in approximately similar abundances when foraging on the inflorescences of centipede grass. In the pan samples, however, *Bombus* spp. and *Apis* spp. were less abundant than *Lasioglossum* spp. in a previous study [12] and the current study. Perhaps this pattern suggests that pan traps are more efficient in collecting halictids than *Bombus* spp. and *Apis* spp. This limitation of pan traps for capturing bees was shown in recent research, in which the authors highlighted that not all approaching bumble bees were captured in the pan traps [25]. In the malaise traps, only *Lasioglossum* spp. were captured; apids were not collected in these traps in the current study, although the exact reason for this finding is not clear.

## 5. Conclusions

The data show that bees and syrphid flies actively forage on centipede grass inflorescences. *Lasioglossum* spp., as well as bumblebees (*Bombus* spp.) were found to directly forage on centipede grass inflorescences. This new knowledge will serve as a foundation for future research, especially when selecting for traits within the centipede grass germplasm that will enhance the foraging activity of bees and the development of bee-friendly turfgrass. It is unclear what role these bees play in the overall pollination of centipede grass. In a previous study on a centipede grass lawn, only pollen from grasses were recovered from *A. mellifera* [17]. Perhaps insect activity on centipede grass inflorescences improves wind pollination by enhancing the release of pollen from the anthers. The data imply that it is critical to conserve bee habitat and encourage bee foraging.

## Figures and Tables

**Table 1:** Details on the study sites and the number of times traps were deployed in each site.

Lawn Site	County in Georgia	Area (m^2^)	GPS Coordinates	Flowering Weeds (%)	No. Visits for Sweep	No. Times Bowl Traps Deployed	No. Times Malaise Traps Deployed	Ave. No. Inflorescences per 929 cm^2^
UGA1	Spalding	946	33.263865, −84.282601	20	5	3	-	16.6
UGA2	Spalding	190	33.267067, −84.292203	0	4	2	2	15.5
UGA3	Spalding	29	33.263220, −84.282771	0	3	-	-	-
Orchard Hill	Spalding	2026	33.197002, −84.220392	15	4	2	2	35.5
New Hope	Pike	1025	33.146915, −84.249020	0	4	2	2	12
William Trail	Pike	2626	33.187282, −84.271020	0	6	3	3	38.3
Zebulon	Pike	1687	33.101468, −84.353520	10	3	2	2	22.5
Countyline	Spalding	716	33.193712, −84.261091	0	2	1	1	10
Cumberline	Spalding	544	33.204567, −84.241432	20	4	2	2	19
Luthersville	Meriwether	1774	33.171635, −84.736221	10	1	1	1	6
Turin	Coweta	1845	33.300773, −84.666347	5	2	2	2	31

**Table 2:** The total number of pollinators collected using various methods.

Sample Method	Family	Genus	No. Pollinators
Sweep			
	Halictidae	*Lasioglossum*	28
	Apidae	*Bombus*	17
	Apidae	*Apis*	9
	Apidae	*Melissodes*	1
	Halictidae	*Augochlorella*	1
	Syrphidae	-	37
Pan			
	Halictidae	*Lasioglossum*	32
	Apidae	*Bombus*	1
	Apidae	*Melissodes*	1
	Halictidae	*Augochlorella*	4
	Syrphidae	-	3
Malaise			
	Halictidae	*Lasioglossum*	7
	Apidae	*Melissodes*	1
